# Women with recurrent spontaneous abortion have decreased 25(OH) vitamin D and VDR at the fetal-maternal interface

**DOI:** 10.1590/1414-431X20176527

**Published:** 2017-09-12

**Authors:** N. Li, H.M. Wu, F. Hang, Y.S. Zhang, M.J. Li

**Affiliations:** 1 Department of Reproductive Medicine, The First Affiliated Hospital of Guangxi Medical University, Nanning, Guangxi, China; 2 Department of Reproductive Medicine, The People's Hospital of Guangxi Zhuang Autonomous Region, Nanning, Guangxi, China

**Keywords:** Recurrent spontaneous abortion (RSA), Treg/Th17 cells, Maternal-fetal interface, 25(OH) D, Vitamin D metabolism

## Abstract

Immunological mechanisms have been proposed to underlie the pathogenesis of recurrent spontaneous abortion (RSA). Vitamin D has a potent immunomodulatory effect, which may affect pregnancy outcome. The objective of this study was to investigate 25-hydroxyvitamin D [25(OH) D] concentration and vitamin D receptor (VDR) expression in the decidual tissues of RSA patients. Thirty women with RSA (RSA group) and thirty women undergoing elective abortion (control group) were recruited during 2016 from gynecology outpatient clinics. We measured 25(OH) D, interleukin (IL)-17, IL-23, transforming growth factor β (TGF-β), VDR and 1-α-hydroxylase (CYP27B1) in decidual tissues collected during the abortion procedure. In the RSA group, 25(OH) D and TGF-β were significantly decreased while IL-17 and IL-23 were significantly increased compared with the control group. VDR expression was significantly decreased in the RSA group compared with the control group. Logistic regression analysis showed a significant negative correlation between 25(OH) D in decidual tissues and RSA. These results indicated that vitamin D concentrations in the decidua are associated with inflammatory cytokine production, suggesting that vitamin D and VDR may play a role in the etiology of RSA.

## Introduction

Recurrent spontaneous abortion (RSA) refers to circumstances where two or more consecutive clinical pregnancies fail before 20 weeks of gestation (1). RSA is a common negative pregnancy outcome that occurs in women worldwide. Causes of RSA are related to genetic factors, anatomical abnormalities, infections, and endocrine disorders (2). However, the causes of the majority of RSA cases are unknown, and may be related to autoimmunity (3).

Vitamin D is a secosteroid hormone that plays a vital role in bone metabolism and mineral homeostasis (4). 1-α-hydroxylase (CYP27B1) and 25-hydroxylase convert vitamin D to its active form, 1,25 dihydroxy-vitamin D_3_ (1, 25(OH)_2_D_3_), which exerts its effect by combining with the nuclear vitamin D_3_ receptor (VDR). In addition to this physiological function, vitamin D modulates the immune system (5), thus vitamin D deficiency/insufficiency could increase the risk of many chronic diseases involving immunological dysfunction (6). The human reproductive process is also regulated by the immune system. Vitamin D has been associated with infertility, polycystic ovary syndrome, *in vitro* fertilization outcomes (7), obstetrical outcomes (8), and male gonadal function (9). During pregnancy, low vitamin D status may increase the risk of obstetrical complications (10). A high proportion of RSA patients reportedly have vitamin D deficiency (11), and low concentrations of vitamin D have been associated with an increased risk of first trimester miscarriage (12). However, the exact effects of vitamin D on pregnancy outcome remain unclear.

Normal pregnancies depend on synchronized immune-endocrine crosstalk at the maternal-fetal interface (13). Recent studies confirm a relationship between CD4^+^ T helper cells (Th) and pregnancy loss. Regulatory T (Treg) cells and T helper type 17 (Th17) cells are important components of the CD4^+^ Th system. Treg cells can secrete transforming growth factor β (TGF-β), play an anti-inflammatory role and maintain the fetal-maternal tolerance during pregnancy. Th17 cells can produce interleukin-17 (IL-17) and play a pro-inflammatory role that has been linked to pregnancy failure. An imbalance in Treg to Th17 cells has been reported in RSA patients (14). Moreover, an increase in Th17 cells and a decrease in Treg cells is reported to increase the risk of RSA (15). Interleukin-23 (IL-23) can promote Th17 cell differentiation and maintain the Th17 response. In a previous study, we found increased IL-17 and IL-23 expression in women with RSA (16). Therefore, it is becoming clear that immune disturbances are involved in the pathogenesis of RSA (17).

As a modulator of the immune system, vitamin D could be implicated in the pathogenesis of pregnancy loss. According to a recent report, vitamin D supplementation significantly down-regulates IL-13 and IL-17 expression in cord blood CD4^+^T cells (18), and regulates the abnormal peripheral cellular immunity of RSA patients (19). Nevertheless, knowledge of how vitamin D affects immune cell function remains inadequate. To our knowledge, there are no reports on the relationship between vitamin D and inflammatory cytokines in decidual tissue, which is an essential part of the maternal-fetal interface. The aim of this study was to measure the concentrations of 25-hydroxyvitamin D (25(OH) D), IL-17, IL-23 and TGF-β, and the expression of CYP27B1 and VDR, in the decidua of women with RSA in the first trimester of pregnancy.

## Material and Methods

### Subjects

Study participants were gynecology clinic outpatients from the First Affiliated Hospital of Guangxi Medical University, Nanning, China, enrolled from January to December 2016. This study was approved by the Ethics Committee of the University. A written informed consent was signed by each participant.

Thirty patients who had experienced two or more consecutive spontaneous abortions during the first trimester of pregnancy were included in the RSA group. The RSA group excluded patients with autoimmune, anatomical, infectious, genetic, or endocrine disorders. The control group included thirty women in early pregnancy who were undergoing artificial miscarriage (elective abortion). The women in the control group had a history of at least one successful pregnancy and no record of abortion or infertility. All study participants were aged 20–35 years, had regular menstrual cycles, and were carrying a single fetus at 7–9 weeks of gestation, as diagnosed with ultrasound. Gestational ages were determined by the first day of the last menstrual period. None of the participants had taken vitamin D or other vitamin supplements for at least 3 months.

In the control group, fetal heart activity was identified during the week before artificial miscarriage. In the RSA group, the patients were recommended to undergo an artificial miscarriage when the fetal heartbeat was not detectable during examination or when it was no longer detectable after being detected previously. Decidual tissues were collected from all participants on the day of the artificial miscarriage operation by negative-pressure aspiration. Decidual tissue samples were dissected into freezing tubes, transported in liquid nitrogen to the laboratory, and stored at −80°C until analysis.

### ELISA for 25(OH) D, IL-17, IL-23 and TGF-β

Decidual tissue samples were washed with precooled phosphate-buffered saline, cut into 1 mm^3^ pieces, homogenized in normal saline (1:9, w/v), and centrifuged at 12,000 *g* for 15 min at 4°C. The liquid supernatant of this tissue suspension was preserved and used for enzyme-linked immunosorbent assay (ELISA). Quantitation of 25(OH) D, IL-17, IL-23 and TGF-β was performed using commercial ELISA kits (catalog numbers CSB-E08097h, CSB-E12819h, CSB-E08461h, and CSB-E04725h, respectively, Cusabio Biotech, China) according to the manufacturer's instructions. The absorbance of each sample was determined at 450 nm using a microplate reader (BioTek, USA).

### Western blotting for VDR and CYP27B1

Western blotting was used to measure the expression of VDR and CYP27B1 in decidual tissues following a previously described method (16). Briefly, total protein was isolated from each decidual tissue sample and mixed with 5× sodium dodecyl sulfate-polyacrylamide gel electrophoresis (SDS-PAGE) loading buffer (Baomanbi, China) at 4:1 v/v. After boiling and cooling, each sample was separated by 10% SDS-PAGE. Samples were then transferred onto a polyvinylidene difluoride (PVDF) membrane (0.45 μm; Millipore Corp., USA) and blocked with 10% skim milk diluted in tris-buffered saline for 2 h at 4°C. PVDF membranes were incubated overnight at 4°C with one of the following antibodies diluted in primary antibody dilution buffer according to the manufacturers' instructions: rabbit anti-VDR (1:1000 dilution; ABclonal Technology Co., Ltd, China), CYP27B1 (1:1000 dilution; Abcam plc., USA), or glyceraldehyde-3-phosphate dehydrogenase (GAPDH; 1:5000 dilution; Proteintech Group, USA). After washing with TBST (TBS containing 0.05% tween), the membranes were incubated for 1 h with a fluorophore-labeled goat anti-rabbit antibody diluted 1:800 in secondary antibody dilution buffer (EarthOX, LLC, USA). A sweep membrane apparatus (LI-COR) was used to detect the signal from each sample. GAPDH was used as the loading control, and quantification was performed using Image J software (National Institutes of Health, USA).

### Statistical analyses

Statistical analyses were performed using SPSS statistical software version 19.0 (SPSS, Inc., USA) and data were graphed using GraphPad Prism software version 5.0 (GraphPad Software, USA). Descriptive data are reported as means±SD. Student's *t*-test was used to compare two groups, Spearman correlation was used to test the correlation between two continuous variables, and a logistic regression model was used to determine independent predictive factors. P<0.05 was considered to be statistically significant.

## Results

### 25(OH) D, IL-17, IL-23 and TGF-β in decidual tissues

**Figure 1.**
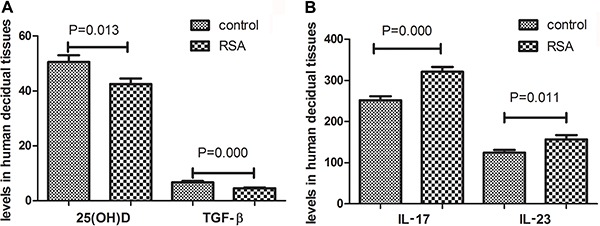
*A*, Concentrations of 25-hydroxyvitamin D (25(OH) D), transforming growth factor β (TGF-β), and *B*, interleukin-17 (IL-17) and interleukin-23 (IL-23) in decidual tissues by enzyme-linked immunosorbent assay, in the recurrent spontaneous abortion (RSA) group compared with the control group. Data are reported as means±SD. Statistical analysis was done with Student's *t*-test.

25(OH) D was significantly decreased in the RSA group (42.49±11.17 µg/L) compared with the control group (50.57±3.18 µg/L). IL-17 and IL-23 were significantly higher in the RSA group than in the control group (IL-17, RSA 320.85±63.15 *vs* control 251.69±51.72 pg/mL; IL-23, RSA 156.73±57.21 *vs* control 124.14±36.60 pg/mL). TGF-β was significantly decreased in the RSA group compared with the control group (RSA 4.52±1.41 *vs* control 6.65±2.81 ng/mL; Figure 1).

**Figure 2.**
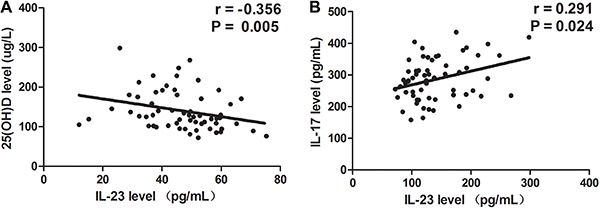
Correlation between 25-hydroxyvitamin D (25(OH) D) and interleukin-23 (IL-23, P=0.005) (*A*), and between interleukin-17 (IL-17) and interleukin-23 (IL-23, P=0.024) (*B*).

Using correlation analysis, 25(OH) D showed a significant negative correlation with IL-23, while IL-23 was significantly positively correlated with IL-17. (Figure 2).

### Expressions of VDR and CYP27B1 by western blotting

**Figure 3.**
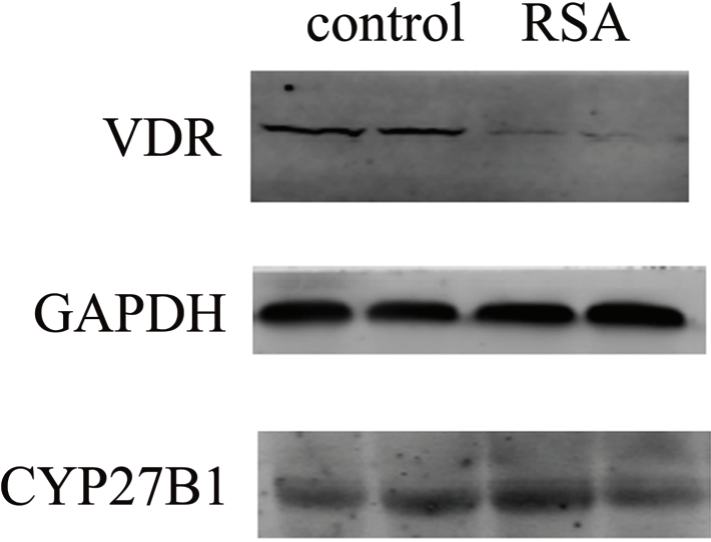
Vitamin D receptor (VDR) and 1-α-hydroxylase (CYP27B1) protein expressions in decidual tissues determined by western blotting. Expression levels were normalized with glyceraldehyde-3-phosphate dehydrogenase (GAPDH). VDR and CYP27B1 were expressed in both the recurrent spontaneous abortion (RSA) and the control groups. VDR was weakly expressed in the RSA group compared to the control group. There was no difference in CYP27B1 expression between the two groups.

The western blotting results showed that VDR expression was significantly decreased in the RSA group compared with the control group, while CYP27B1 expression showed no significant difference between the two groups (Figure 3).

### Correlation between RSA and 25(OH) D

In order to obtain the predictive risk of RSA, we analyzed the correlation between 25(OH) D in decidual tissues and RSA to determine the predictive strength of the former for RSA. A logistic regression analysis showed that RSA was significantly negatively correlated with 25(OH) D (Exp (B)=0.945, P=0.019), indicating that lower concentrations of 25(OH) D in the decidual tissues are associated with a higher risk of RSA.

## Discussion

To our knowledge, this is the first study investigating 25(OH) D concentration together with VDR and CYP27B1 expression in the decidual tissues of RSA and normal pregnancies. The main findings of this study were that 25(OH) D and TGF-β concentrations and VDR expression were significantly decreased, and IL-17 and IL-23 concentrations were significantly increased in women with RSA compared to women with normal pregnancies. Our study is the first to show a negative relationship between 25(OH) D and the IL-23/IL-17 axis localized at the maternal-fetal interface.

During pregnancy, systemic and peripheral immunomodulation of the mother is vital for the acceptance of the fetus, which is a semi-allograft. Although they have opposed profiles, the balance of Treg/Th17 cells has been shown to be important to the persistence of pregnancy (20). A decrease in Treg cells and/or an increase in Th17 cells plays a critical role in the pathogenesis of RSA (15). IL-23 contributes to the proliferation and differentiation of Th17 cells, and promotes the secretion of IL-17, making up the so-called IL-17/IL-23 axis. Our previous studies and others have discovered higher concentrations of IL-23 in RSA patients (16,21). In this study, we demonstrated that TGF-β is decreased while IL-17 and IL-23 are increased in the decidual tissues of women with RSA, findings that are in agreement with the aforementioned references (15,16). Therefore, the function of the IL-17/IL-23 axis at the maternal-fetal interface could be important to the immunopathology of RSA.

In recent years, the importance of the immunological role played by vitamin D at the fetal-maternal interface has been increasingly recognized (4); likewise, its role in the pathogenesis of RSA has also begun to attract attention. 25(OH) D is the best indicator of vitamin D nutritional status in clinical practice because of its long half-life and relative stability (22). A recent study (23) reported that serum 25(OH) D concentrations are higher in women with normal pregnancies than in those with first trimester miscarriages, suggesting that vitamin D deficiency is associated with pregnancy loss. However, the maternal-fetal interface is where direct contact occurs between mother and fetus, and aspects of its local microenvironment, which is crucial for maintaining normal pregnancy, may differ from those of the serum. Therefore, we considered it important to investigate 25(OH) D concentrations in decidual tissues. Here, we observed that 25(OH) D concentrations in the RSA group were decreased compared with the control group, suggesting that an altered localized maternal vitamin D state may influence pregnancy outcome. Therefore, this study shows that the 25(OH) D concentration in decidual tissues (as well as that in serum) is decreased in women with RSA compared with those with normal pregnancies.

As an immune modulator, vitamin D can up-regulate the expression of VDR in activated T cells, up-regulate Treg cell production, and down-regulate Th17 cell production (24). Dendritic cells are critical to the differentiation of Th17 cells, and vitamin D can modulate dendritic cell maturation and indirectly inhibit the proliferation of Th17 cells. Vitamin D can also inhibit the Th17-specific transcription factors retinoic acid orphan receptor γt (RORγt) and IL-23R and inhibit the generation of the Th17 cells (25). Moreover, vitamin D can suppress the secretion of IL-17 polarized cytokines such as IL-2 and IL-6, thereby inhibiting the action of Th17 cells (26), and can enhance Treg cell number and function (27). Thus, vitamin D might play a role in the balance between Treg and Th17 cells and affect the risk of RSA.

Knowledge of the relationship between Treg/Th17 cell imbalance and vitamin D concentration in the pathogenesis of RSA is, however, limited. Previous reports indicate that the role of vitamin D in regulating immune function is similar to that of IL-10, and vitamin D could act as an immunomodulatory agent in preparation for *in vitro* fertilization pre-embryo transfer or for prevention or treatment of RSA, preeclampsia or eclampsia (28). In our published research, we report that IL-23 promotes the differentiation of Th17 cells while an anti-IL-23 antibody promotes the differentiation of Treg cells. IL-23 may thus hold potential as a target for RSA treatment (29). In view of previous research, we postulated that vitamin D might play a role in the pathogenesis of RSA via IL-23. This study was therefore designed to explore the relationship between vitamin D and the IL-23/IL-17 axis at the fetal-maternal interface. We found that low 25(OH) D concentrations in decidual tissues were associated with RSA, and that 25(OH) D was negatively correlated with IL-23, suggesting that vitamin D might play a vital role in influencing the IL-23/IL-17 axis. Decreased vitamin D concentrations might be one of the causes of the Treg/Th17 imbalance leading to abortion. Moreover, results of a double-blind randomized controlled trial examining the effect of vitamin D supplementation on RSA showed that this supplementation decreased serum IL-23 and the incidence of abortion (30).

We then further investigated vitamin D metabolism in decidual tissues. Vitamin D plays an important role promoting crosstalk between the maternal decidua and fetal trophoblast. Higher vitamin D concentrations may promote successful pregnancy development (31). The placenta is one of the main sites of extra-renal conversion of 25(OH) D to its active form, and CYP27B1 and VDR are expressed in human decidua and placenta (32). However, it is unclear whether the expression of VDR and CYP27B1 differs between RSA patients and women with normal pregnancies. In this study, we found that both VDR and CYP27B1 are expressed in the decidua of women from the RSA and control groups, with VDR expression significantly reduced in the RSA group and no difference in CYP27B1 expression between groups. The presence of VDR and CYP27B1 in decidual tissues is indicative of vitamin D metabolism and autocrine regulatory signaling at the maternal-fetal interface (33). As an immune modulator, vitamin D action depends on VDR. Localized areas of low 25(OH) D may lead to a shortage of vitamin D_3_. Reduced VDR expression and/or vitamin D_3_ insufficiency might affect the downstream signaling pathway of vitamin D in decidua, leading to a poor pregnancy outcome.

In conclusion, our study provides experimental evidence of an association between RSA and vitamin D at the fetal-maternal interface. Our RSA group had low 25(OH) D concentrations and reduced VDR expression in decidual tissues, suggesting that low concentrations of 25(OH) D and VDR might be associated with RSA. Our findings suggest that vitamin D localized at the fetal-maternal interface had a protective role against pregnancy loss. Larger studies are required to validate these conclusions. Studies investigating the effect of vitamin D on the balance of Treg/Th17 in early pregnancy, perhaps using *in vitro* cell culture methods or animal models, would also be informative, as well as rigorous clinical studies on vitamin D supplementation in RSA patients.
